# Freeze-drying of mammalian cells using trehalose: preservation of DNA integrity

**DOI:** 10.1038/s41598-017-06542-z

**Published:** 2017-07-24

**Authors:** Miao Zhang, Harriëtte Oldenhof, Bulat Sydykov, Judith Bigalk, Harald Sieme, Willem F. Wolkers

**Affiliations:** 1 Institute of Multiphase Processes, Leibniz Universität Hannover, Hannover, Germany; 2 Unit for Reproductive Medicine, University of Veterinary Medicine Hannover, Hannover, Germany

## Abstract

The aim of this study was to investigate preservation of biomolecular structures, particularly DNA, in freeze-dried fibroblasts, after loading with trehalose via freezing-induced uptake. Cells were freeze-dried with trehalose alone or in a mixture of albumin and trehalose. Albumin was added to increase the glass transition temperature and storage stability. No viable cells were recovered after freeze-drying and rehydration. FTIR studies showed that membrane phase behavior of freeze-dried cells resembles that of fresh cells. However, one day after rehydration membrane phase separation was observed, irrespective of the presence or absence of trehalose during freeze-drying. Freeze-drying did not affect the overall protein secondary structure. Analysis of DNA damage via single cell gel electrophoresis (‘comet assay’) showed that DNA damage progressively increased with storage duration and temperature. DNA damage was prevented during storage at 4 °C. It is shown that trehalose reduces DNA damage during storage, whereas addition of albumin did not seem to have an additional protective effect on storage stability (i.e. DNA integrity) despite the fact that albumin increased the glass transition temperature. Taken together, DNA in freeze-dried somatic cells can be preserved using trehalose as protectant and storage at or below 4 °C.

## Introduction

Lyophilization is one of the most widely used methods for dry preservation of biological materials^1^. Freeze-drying has been applied for preservation of proteins and liposomes for pharmaceutical applications, using sugars as the main protectant^2–4^. Freeze-drying of cells, however, is less frequently done due to difficulties to load cells with lyoprotectants. Cells which are inherently more resistant towards drying stress such as bacteria^5^ and yeast^6^ synthesize lyoprotectants upon exposure to stress and can be freeze-dried, while resuming metabolism upon rehydration. Mammalian cells typically do not survive drying but biomolecules are often well preserved. Sperm chromatin structure in freeze-dried sperm for example, is largely intact and can be used to fertilize an oocyte by means of intracytoplasmic sperm injection^7^. Freeze-dried platelets retain clotting properties and can be used for topical wound healing^8, 9^. Other studies have shown the potential of freeze-drying to preserve genetic information of somatic and stem cells^10–12^.

During freeze-drying, samples are first frozen, after which ice is removed via sublimation under vacuum. During the secondary drying phase, residual moisture of the sample is reduced to water contents around 0.05 g H_2_O per g dry weight or lower. The entire freeze-drying process involves drastic changes in sample temperature, hydration level, and pressure conditions. Both freezing and drying are severe stress factors which can be damaging to biomolecules and cellular structures^13^. Especially removal of water surrounding biomolecules may lead to irreversible structural changes in phase state and organization of cellular membranes and protein aggregation^14^. In addition, increased levels of reactive oxygen species (ROS) are known to cause damage. Lipids in membranes are especially sensitive to free radical attack by ROS^15^. Despite that no viable cells are recovered after freeze-drying, chromatin is often well preserved, and nuclei of freeze-dried cells can be transferred into other cells^10, 16, 17^. However, chromatin is subject to oxidative attack during storage^18, 19^.

Freeze-drying requires use of specific protectants to stabilize biomolecules during both freezing and drying. Examples include non-reducing disaccharides such as sucrose and trehalose. These sugars have good glass-forming properties^20, 21^, and can replace hydrogen bonds of water with biomolecules upon dehydration^22^. A glass is a highly viscous state in which cellular structures are embedded while simultaneously molecular mobility and damaging reactions are slowed down^23–25^. Glasses composed of single compounds or mixtures display a characteristic glass transition temperature below which viscosity drastically increases. The glass transition temperature (T_g_) of sugars is dependent on the molecular weight of the sugar as well as intermolecular interactions. Generally, the T_g_ of sugars increases with increasing molecular weight. Among the disaccharides, trehalose has an anomalously high glass transition temperature of nearly 60 °C higher than that of sucrose which has the same molecular weight. Macromolecules, such as albumin and hydroxyethyl starch, can be added to freeze-drying formulations to increase the T_g_ and storage stability^26–28^. Water acts as a plasticizer and decreases the glass transition temperature of freeze-dried samples. The glass transition temperature and hence storage stability is dependent on the residual moisture content after freeze-drying^29^.

One of the challenges with using sugars for freeze-drying of cells is to load the cells with sugars for intracellular protection^8, 30^. We have recently shown that cells actually take up trehalose if exposed to freezing. This occurs by membrane imperfections that are caused by freezing-induced membrane phase transitions^31, 32^. In a variety of studies, we have shown that freezing-induced trehalose uptake coincides with good cryosurvival of cells^31, 33, 34^.

The aim of this study was to investigate intactness of biomolecular structures, particularly DNA, in freeze-dried fibroblasts, after loading the cells with trehalose during freezing. Cells were freeze-dried in formulations composed of sugars and albumin with known differences in glass transition temperature. The freeze-drying formulations were first characterized in terms of their glassy properties. Membrane lipid phase behavior and the overall protein secondary structure were studied using Fourier transform infrared spectroscopy. DNA damage in freeze-dried cells was studied during storage at various temperatures using the ‘comet assay’.

## Results

### Glass transition temperatures and hydrogen bonding interactions of sugar/albumin glasses

**Figure 1:**
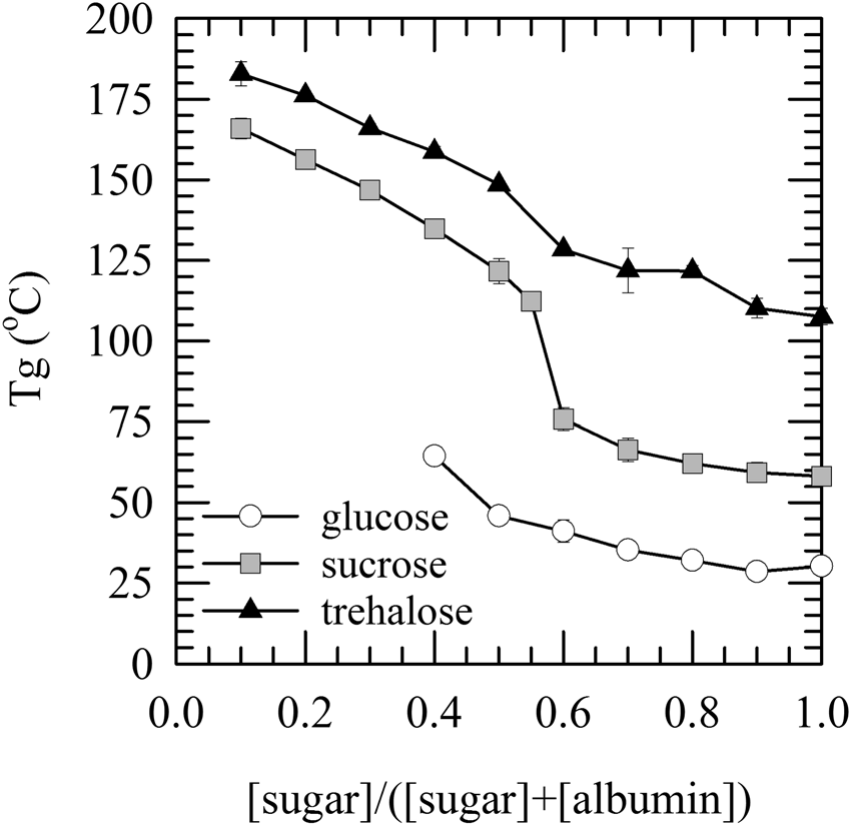
Glass transition temperature of sugar/albumin formulations. Glasses were prepared by drying mixtures of albumin and the disaccharides trehalose (black triangles) and sucrose (grey squares) as well as the monosaccharide glucose (open circles). Glasses had a water content of less than 0.05 g H_2_O per g dry weight^−1^. FTIR was used to derive the glass transition temperature (T_g_). The dependency of T_g_ as a function of the relative sugar mass content is shown for the various sugar/albumin mixtures. Mean values ± standard deviations were determined from at least three independent experiments.

The glass transition temperature is one of the most important parameters determining storage stability of lyophilized materials. FTIR was used to study glass transition temperatures of trehalose/albumin mixtures, which were used for freeze-drying of fibroblasts. Mixtures of sucrose and glucose with albumin were studied for comparison. Glassy behavior of dry sugar/albumin mixtures was analyzed using infrared spectroscopy. Figure 1 shows how albumin addition affects the glass transition temperature (T_g_). T_g_ values for pure glucose, sucrose, trehalose, and albumin glasses were determined to be 30 ± 1, 58 ± 1, 108 ± 3, and 180 ± 4 °C, respectively. It can be seen that T_g_ increases with increasing albumin content for all sugars tested. Trehalose/albumin mixtures have the highest T_g_ values. At a sugar content of 0.5 an inflection point is seen in the relationship between T_g_ and the sugar content for both sucrose and trehalose. No glass transition was observed in glucose/albumin mixtures at glucose contents in the mixture below 0.4 likely due to reactions between the reducing glucose and albumin (browning reactions) during heating.

### Water contents and glass transition temperatures of freeze-dried cell samples

Sample water contents and T_g_ values of freeze-dried fibroblasts are listed in Table 1. Cells suspended in saline medium (HSKM) without protective agents contained a higher residual moisture content (0.12 ± 0.02 g H_2_O g DW^−1^) as compared to samples freeze-dried with protectants (0.06 ± 0.01 and 0.01 ± 0.00 g H_2_O g DW^−1^ for trehalose and trehalose/albumin, respectively). HSKM without protective additives formed a uniform porous structure after freeze-drying, however, no glass transition was observed with DSC analysis. Glass transition temperatures were evident in samples freeze-dried with trehalose or trehalose/albumin with T_g_ values of 19 ± 6 °C and 97 ± 8 °C, respectively.

**Table 1:** Sample moisture contents and T_g_ values of dried samples, as determined directly after freeze-drying.

	water content (g H_2_O per g dry weight)	T_g_ (°C)
HSKM	0.12 ± 0.02	—
HSKM + TRE	0.06 ± 0.01	18.9 ± 6.3
HSKM + TRE/BSA	0.01 ± 0.00	97.2 ± 8.0

Cells were suspended in saline medium (HSKM) without protective agents, as well as supplemented with trehalose (250 mM) or trehalose/albumin (1/1 mass ratio, 9.5% each); and subjected to freeze-drying as described in detail in the methods section. Water contents were determined gravimetrically, and glass transition temperatures were determined using DSC. Mean values ± standard deviations were determined from four independent experiments.

### Cell survival after freezing and freeze-drying with trehalose

Fibroblasts were cryopreserved and freeze-dried in HSKM medium without protectants as well as medium supplemented with trehalose, and cell viability was analyzed. Addition of 250 mM trehalose before freezing, did not affect percentages of membrane intact cells (94 ± 4%). Samples cryopreserved with trehalose showed 62 ± 20% membrane intact cells after thawing, whereas in the absence of trehalose 37 ± 15% membrane intact cells were recovered showing trehalose has cryoprotective properties. After freeze-drying, however, no membrane intact cells were recovered, irrespective of the addition or absence of trehalose (1 ± 1 and 0%, respectively).

### Infrared spectra of fibroblasts

**Figure 2:**
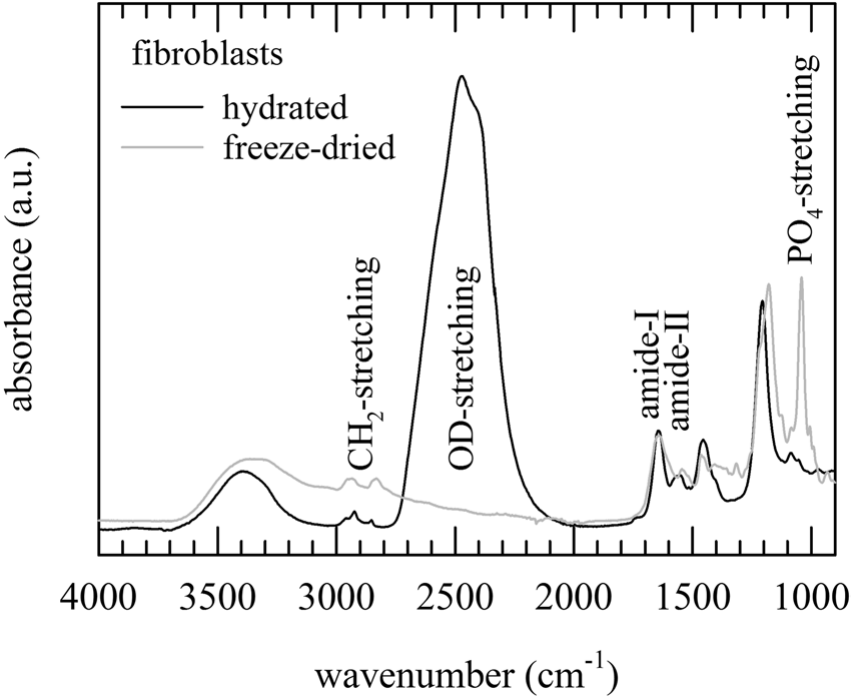
*In situ* infrared spectra of fibroblasts; both, ATR-FTIR spectra of freeze-dried samples without protectants (grey line) and hydrated samples in saline/D_2_O (black line) are shown. The full spectral region (4000–900 cm^−1^) is presented, with indicated absorbance bands arising from endogenous membrane lipids (CH_2_-streching bands, 3000–2800 cm^−1^), proteins (amide-I and -II bands, 1700–1500 cm^−1^), and (nuclear) DNA (PO_4_-streching bands, 1300–1000 cm^−1^). The OD-stretching band arising from D_2_O is located between 2700–2200 cm^−1^.

Infrared spectroscopy was used to evaluate preservation of membranes and proteins in freeze-dried fibroblasts. Figure 2 shows spectra of hydrated as well as freeze-dried fibroblasts. Hydrated samples were prepared in saline/D_2_O to avoid interference of the H_2_O band with the protein amide-I band. The OD-stretching band can be seen around 2500 cm^−1^. The CH_2_-streching vibration bands predominantly arising from the membrane lipid acyl chains are seen in the 2900–2800 cm^−1^ region. The amide-I and -II bands arising from endogenous proteins, are seen at 1640 and 1560 cm^−1^, respectively. The 1300–900 cm^−1^ region is designated as the fingerprint region.

### Membrane phase behavior

**Figure 3:**
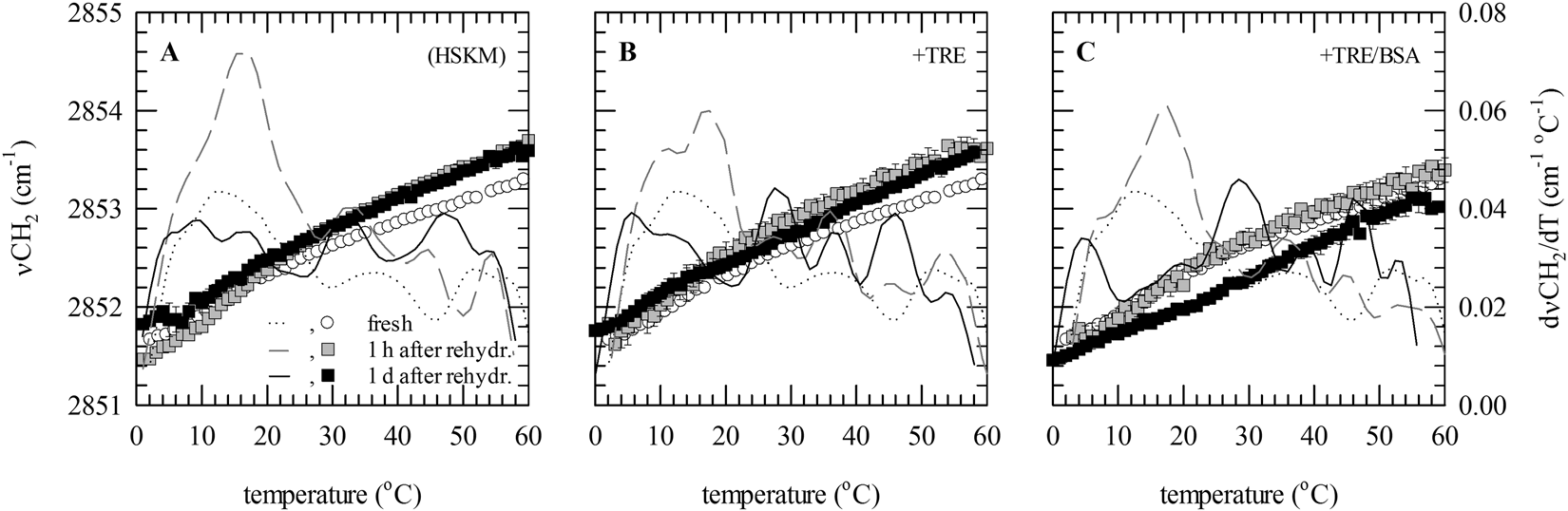
Membrane phase behavior of fresh fibroblasts (dotted lines, open circles) as well as fibroblasts that were freeze-dried without protectants (**A**), with trehalose (**B**) or trehalose/albumin **(C**). Freeze-dried samples were analyzed directly after rehydration (grey lines, grey squares) as well as 1 d after rehydration (black lines, closed squares). FTIR transmission spectra were collected during heating of a sample from 0 to 80 °C at 2 °C min^−1^, the position of the CH_2_-stretching vibration band (*v*CH_2_) arising from membrane lipids was determined and plotted as a function of the sample temperature (circles and squares). First derivatives were determined to visualize phase transitions more clearly (lines). Mean values ± standard deviations were determined from three independent experiments.

Membrane phase behavior of freeze-dried fibroblasts was evaluated directly after rehydration as well as 1 d after rehydration. Fibroblasts were freeze-dried without protectants, with trehalose or with trehalose/albumin. Membrane phase behavior was studied by monitoring the CH_2_ band arising from membrane lipids as a function of the sample temperature. First derivatives were calculated to visualize membrane phase transitions more clearly (Fig. 3). Fresh controls, exhibited a non-cooperative broad transition from 10–15 °C. After freeze-drying, multiple lipid melting events were seen irrespective of the absence or presence of trehalose and/or albumin. Directly after freeze-drying, a main phase transition was seen at ~17 °C, whereas after 1 d additional lipid melting events were seen at ~6 and ~27 °C. This could denote membrane phase separation caused by accumulation of lipid peroxidation products altering lipid phase behavior.

### Overall protein secondary structure

**Figure 4:**
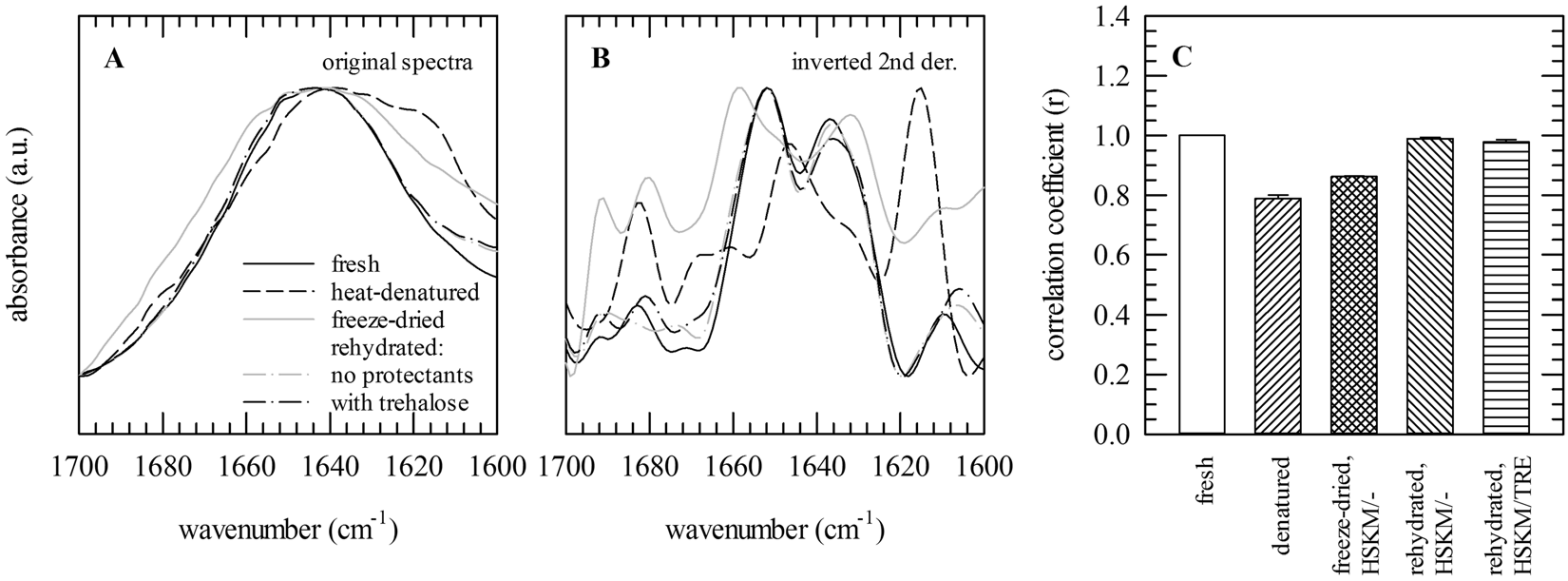
*In situ* ATR-FTIR spectra of fibroblasts in the 1700–1600 cm^−1^ spectral region, containing amide-I band arising from endogenous proteins. Normalized original spectra (**A**) are shown, as well as inverted second derivative spectra (**B**) to reveal differences more clearly. The correlation coefficient (r) was calculated to quantify differences in secondary structure between fresh cells and the different treatment groups (**C**). Correlation coefficients of fresh (solid black lines, white bars) and dried fibroblasts (solid grey lines, bars with diamonds) are shown; both in saline solution (HSKM). Furthermore, data are shown for rehydrated fibroblasts after freeze-drying without protectants (dash-dotted grey lines, bars with downward diagonals) or with trehalose (dash-dotted black lines, bars with horizontal lines). For comparison, data of heat-denatured samples (dashed black lines, with upward diagonals) are shown.

Figure 4 shows the amide-I band regions (1700–1600 cm^−1^) of fresh and freeze-dried fibroblasts, both before and after rehydration. For comparison, spectra of heat-denatured cells are presented. The amide-I band profile of rehydrated cells closely resembles that of fresh cells. In the dried state the amide-I band profile differs from that of hydrated and rehydrated cells. Heat denatured samples show pronounced bands at 1620 and 1684 cm^−1^ indicating extended β-sheet structures. No signs of protein denaturation were observed in rehydrated cells irrespective of the presence of trehalose. Correlation coefficients (r-values) were calculated to quantify differences in amide-I band profile compared to that of fresh cells (Fig. 4C). The correlation coefficient of heat-denatured cells was determined to be 0.78, denoting structural changes in overall protein structure upon denaturation. In the dried states r-values were determined to be 0.89 and 0.86 for sample freeze-dried with and without trehalose, respectively. After rehydration, however, r-values of 0.98 and 0.99 were found for samples freeze-dried with and without trehalose, respectively.

### DNA stability of freeze-dried cells during storage

**Figure 5:**
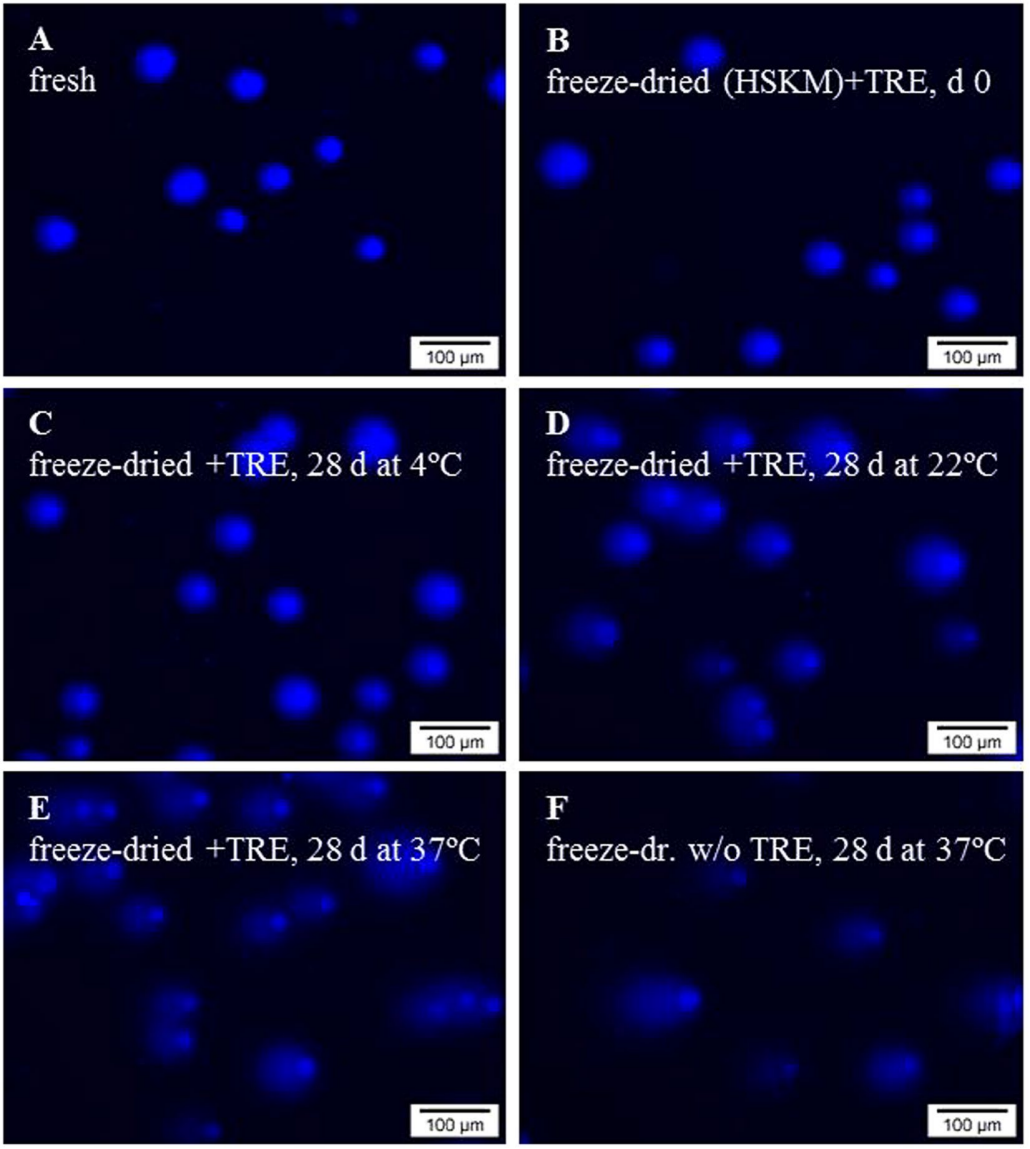
Micrographs acquired with the ‘comet assay’ for assessing chromatin integrity and DNA damage; for fresh fibroblasts (**A**) as well as fibroblasts which were subjected to freeze-drying with trehalose (**B**–**E**) or without protective agents (**F**) and different storage conditions in the dried state. Freeze-dried specimens were rehydrated and analyzed directly after freeze-drying (**B**) as well as after 28 d storage at 4 °C (**C**), 22 °C (**D**) and 37 °C (**E**,**F**). Cells embedded in agarose were lysis-treated and subjected to alkaline electrophoresis, after which they were stained with the DNA intercalating dye Hoechst. Increased DNA damage is evident as longer ‘comet tails’ and increased fluorescence intensity in the ‘tail’ at expense of the ‘head’ or nuclear region intensity.

Chromatin integrity and DNA damage of fibroblasts were determined before and after freeze-drying and storage for up to 1 month at different temperatures using the ‘comet assay’. This assay involves Hoechst-staining of DNA after lysis treatment and gel electrophoresis for separation of DNA fragments according to their lengths and extent of damage. Representative microscopic images are shown in Fig. 5. Intact nuclear DNA is present in a comet ‘head’, whereas fragmented DNA appears as a ‘tail’. Fresh fibroblasts exhibit intensive fluorescence predominantly in the comet head and little fluorescence in the tails. DNA damage during storage is evident as an increased fluorescence in the comet tail at expense of fluorescence in the head. In addition, the length of the comet tail is longer after storage at elevated temperatures, and if freeze-drying is done without trehalose.

**Figure 6:**
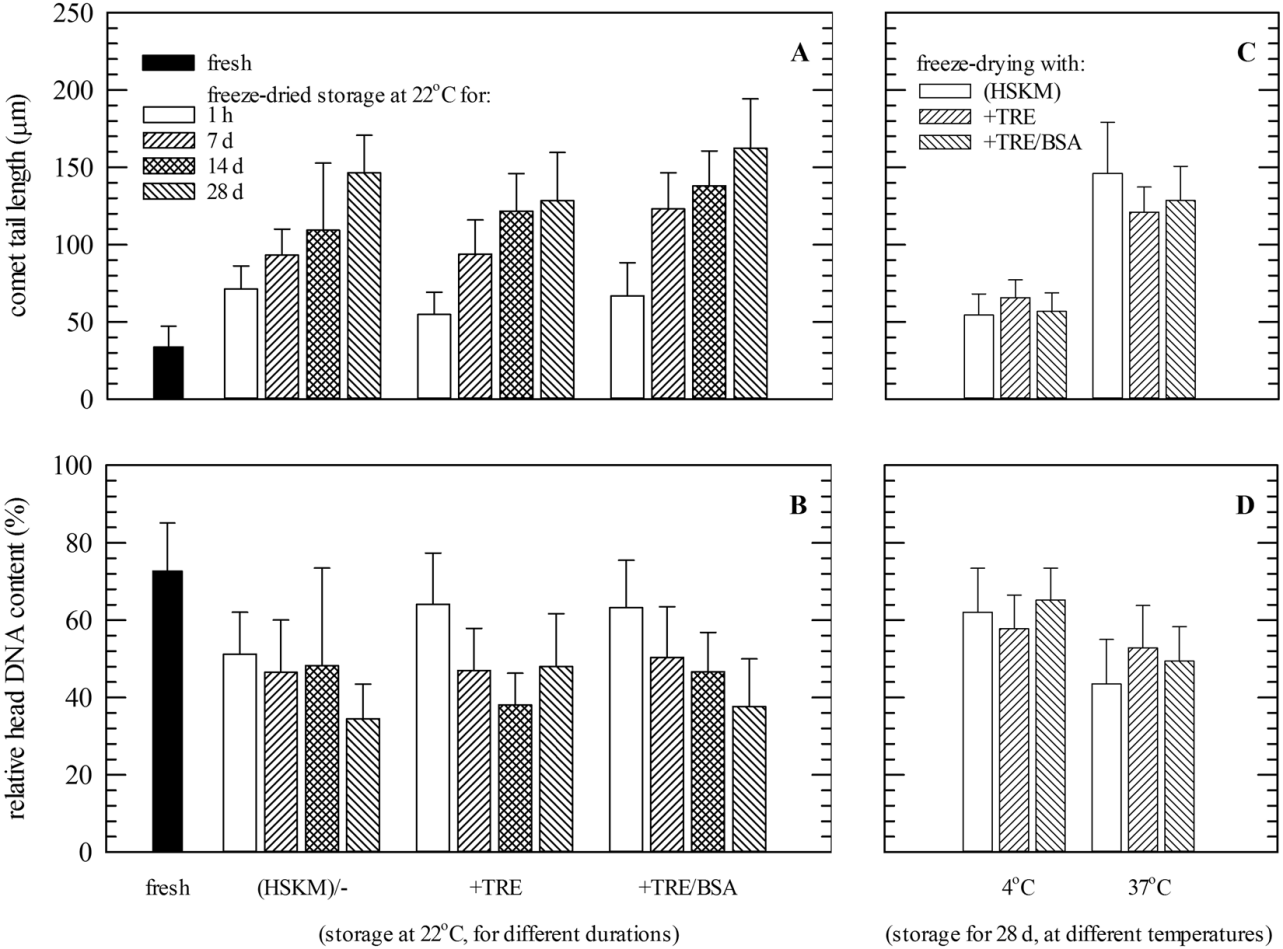
DNA damage during dried storage of fibroblasts was evaluated using the ‘comet assay’. Fibroblasts were freeze-dried in saline solution without supplements (HSKM) or with trehalose or trehalose/albumin. Storage was done for different durations at room temperature (**A**,**B**) as well as at different temperatures for 1 month (**C**,**D**). Analysis was done prior to freeze-drying (black bars), directly after freeze-drying (white bars) and after 1–4 weeks storage at room temperature (7 d: bars with upward diagonals, 14 d: bars with diamonds, 28 d: bars with downward diagonals) (**A**,**B**). Furthermore, specimens were analyzed after 28 d storage at 4 °C and 37 °C (without supplements: white bars, with trehalose: bars with upward diagonals, with trehalose/albumin: bars with downward diagonals) (**C**,**D**). From microscopic images of Hoechst-stained specimens, both the comet tail lengths were determined (**A**,**C**) and relative DNA contents (i.e. Hoechst fluorescence) in comet heads versus tails (**B**,**D**). Mean values ± standard deviations were determined from two independent experiments, and a minimum of 30 ‘comets’ were analyzed per treatment.

‘Comets’ were analyzed to quantify DNA damage during storage. Figure 6 shows comet tail lengths and relative DNA contents (fluorescence intensity) in the heads versus tails. After freeze-drying comet tails were found to be increased for all formulations tested (55–71 versus 34 ± 13 μm for non-treated controls). Comet tail lengths progressively increase during storage at room temperature indicating a loss of DNA integrity. Freeze-drying with trehalose resulted in less DNA damage (i.e. shorter tails of 128 ± 31 μm at d 28) compared to samples which were freeze-dried without protectants or with trehalose/albumin (146 ± 24 and 158 ± 41 µm, respectively; p < 0.05). Differences were already seen after 2 weeks. Corroborating trends were observed in relative DNA contents in the comet head/tail. The relative DNA content in the tail progressively increases during storage at room temperature. During storage at 37 °C comet tails were significantly shorter if trehalose was used for freeze-drying, whereas addition of albumin did not seem to have additional protective effects. At 4 °C, comet tail lengths as well as DNA contents in the head after 4 weeks storage did significantly differ compared to those directly after freeze-drying (Fig. 6C and D).

## Discussion

Chromatin in freeze-dried somatic cells is intact directly after freeze-drying, but subject to damage during storage. DNA damage progressively increases with storage duration and temperature. Trehalose reduces DNA damage during storage, whereas addition of albumin does not have additional protective effects on DNA storage stability.

For dry preservation of biomaterials, disaccharides like sucrose and trehalose are used instead of glucose^4, 20, 35^, because sucrose and trehalose have a higher glass transition temperature compared to glucose^36, 37^ and do not have an open form with a free aldehyde group which can react with proteins. Addition of albumin increases the T_g_. Therefore, addition of a protein/polymer like albumin to sugar formulations for dry preservation of cells has been suggested to improve storage stability^8, 38^. However, we did not find beneficial effects of albumin for preserving chromatin in freeze-dried fibroblasts.

The T_g_ of samples freeze-dried with trehalose and albumin is clearly higher than that of sample freeze-dried with trehalose only. It should be noted, however, that the T_g_ mostly reflects the extracellular matrix and not the intracellular T_g_. In a variety of studies we have shown that trehalose enters the cell during freezing-induced membrane phase transitions^31, 33, 34^. By contrast, the much larger albumin likely does not enter the cell during freezing-induced phase transitions. So despite the fact that the albumin increases the T_g_ of the freeze-drying formulation, the intracellular T_g_ is not necessarily increased when albumin is used. This could explain the fact that albumin does not improve cellular DNA storage stability in the current study. Albumin is still useful as bulking agent in the freeze-drying formulation because it separates cells in the freeze-dried matrix. For platelets, we have shown that cell recovery increases when a combination of trehalose and albumin is used for freeze-drying instead of trehalose alone^8^. One of the advantages of using albumin is that the freeze-dried cake has a better appearance and is more stable (i.e. no collapse), and is easier to dissolve in water compared to using trehalose alone.

After freeze-drying, no membrane intact cells were recovered, irrespective of the use of protective agents. Also for other cell types, typically no membrane intact cells are recovered after freeze-drying^10, 16, 17^. Membrane phase behavior of rehydrated cells resembles that of fresh cells. However, multiple distinct melting peaks were seen in rehydrated cells which became more pronounced 1 d after rehydration. This likely reflects membrane phase separation caused by accumulation of phospholipid peroxidation products (i.e. lysophospolipids). The latter may result from high levels of ROS formed during freezing and/or drying^39, 40^. The overall protein secondary structure was affected by drying. However, after rehydration the overall protein secondary structure closely resembled that of fresh cells.

Preservation of chromatin in somatic cells is of interest for applications were nuclear material of freeze-dried cells is transferred to other cells^41^ or for amplification of specific sequences. It was found here that freeze-drying resulted in some DNA degradation, which is reduced in the presence of trehalose. DNA damage progressively increased during storage at room temperature. Trehalose cannot prevent DNA damage during room temperature storage, but the damage is less than in the absence of trehalose. Free radical-mediated oxidation has been implicated as the main cause of DNA degradation during storage^42, 43^, irrespective of the presence of a glassy state^18^. ROS that have accumulated during freeze-drying may directly alter chromatin structure upon rehydration. In addition, lipid peroxidation products may damage DNA^44, 45^. DNA stability in dried samples is determined by the residual moisture content of the sample and the presence or absence of lyoprotectants^46, 47^. Residual water accelerates degradation reactions. In addition, environmental factors such as temperature, relative humidity, and oxygen levels are known to affect DNA stability^48^. Generally, DNA packaged in chromatin is more stable than naked DNA and purified DNA is more stable than DNA in cells or tissues due to the presence of oxidizing compounds^48^. It has been shown that unsaturated lipids facilitate oxidative DNA damage in lyophilized lipoplexes during storage^18^.

In conclusion, despite no viable cells were recovered after freeze-drying and rehydration of fibroblasts, biomolecular structures appear to be largely intact. Directly after freeze-drying, membrane phase behavior of freeze-dried cells resembles that of fresh cells and freeze-drying did not affect the overall protein secondary structure. Chromatin is intact after freeze-drying, but is subject to damage during storage. It is shown that DNA damage progressively increases with storage duration and temperature and that trehalose reduces DNA damage during storage. Even though addition of albumin increased T_g_, it did not seem to have a positive effect on DNA storage stability. This likely can be attributed to a lack of intracellular albumin, which means that the intracellular T_g_ is not increased in the presence of albumin. Accumulation of DNA damage in freeze-dried cells during storage can be prevented by storing samples at or below 4 °C.

## Materials and Methods

### Cell culture conditions

Mouse embryonic fibroblast (3T3) cells were grown in Dulbecco’s modified Eagle medium (DMEM) supplemented with 10% (v/v) fetal bovine serum and 100 units mL^−1^ penicillin/streptomycin (Biochrom, Berlin, Germany). Cultures were grown at 37 °C in the presence of 5% CO_2_, in T-75 flasks (TPP Techno Plastic Products AG, Trasadingen, Switzerland). Every 2–3 days, cells were trypsinized via incubation with 0.05% (v/v) trypsin for 3 min at 37 °C, followed by dilution in fresh DMEM. Then, cell suspensions were centrifuged (8 min at 1000 × g), the supernatant was aspirated and the pellet was re-suspended at 0.75 × 10^6^ cells mL^−1^ with fresh DMEM.

### Cryopreservation and freeze-drying

Cell pellets were diluted to 25–35 × 10^6^ cells mL^−1^ in HEPES buffered saline (HSKM; 10 mM HEPES, 137 mM NaCl, 2.7 mM KCl, 0.5 mM MgCl, pH 7.4), followed by slowly adding an equal volume of HSKM supplemented with two-fold the desired final concentration of protective agents; resulting in 200 μL 12.5–17.5 × 10^6^ cells mL^−1^. Trehalose (TRE; Pfanstiehl, Zug, Switzerland) and bovine serum albumin (BSA, MW: 67000 g mol^−1^; Serva, Heidelberg, Germany) were used as protective agents. Trehalose was tested alone at a 250 mM final concentration, and in combination with an equal mass of albumin (1/1 mass ratio; 9.5% w/v each). Samples were transferred into freeze-drying vials (Christ 2 R injection vials; Landgraf Laborsysteme, Langenhagen, Germany), and frozen in a controlled rate freezer (CM-2000; Carburos Metalicos, Madrid, Spain) at 40 °C min^−1^ down to −80 °C. Then, samples were plunged into liquid nitrogen and stored at −150 °C, for a minimum of 24 h, in a freezer (MDF-1155ATN; Sanyo Electric Biomedical Co., Bad Nenndorf, Germany). For analysis after cryopreservation, samples were thawed during 5 min in a 37 °C water bath, and analyzed within 1 h. Freeze-drying was done by transferring frozen samples to the temperature-controlled shelves of a lyophilizer (Virtis Advantage Plus freeze-dryer; SP scientific, Warminster, USA). The initial shelf temperature was set at −30 °C, after which samples were cooled to −40 °C and kept at this temperature for 60 min while the chamber pressure was decreased to 60 mTorr. Primary drying was done at −30 °C for 400 min. Secondary drying was done by increasing the shelf temperature to 40 °C at 0.1 °C min^−1^, after which the shelf temperature was decreased again to room temperature (20 °C). Rehydration was done by adding 200 µL distilled water to the dried samples in a drop-wise fashion, and survival was determined within 1 h after rehydration.

Water contents (WC; in g H_2_O per g dry weight) of freeze-dried samples were determined by comparing the fresh weight (FW; in g, measured directly after freeze-drying) and dry weight (DW; in g, measured after overnight incubation in an oven at 80 °C).

### Cell viability measurements

Cell concentrations and percentages of membrane intact cells were determined via trypan blue staining and using a hemocytometer (Neubauer-Improved; Assistent, Sondheim v. d. Rhön, Germany) under a light microscope (Axiovert 40 C; Carl Zeiss, Göttingen, Germany). Samples (200 μL) were diluted with HSKM or HSKM supplemented with protective agents (800 μL). Relative numbers of membrane intact/damaged cells were scored (i.e. not stained or exhibiting intracellular trypan blue staining) and cell concentrations were determined.

### Glass transition temperature (T_g_) measurements using differential scanning calorimetry

Differential scanning calorimetry (DSC) measurements were performed using a Netzsch DSC 204F1 Phoenix instrument (Netzsch-Gerätebau GmbH, Selb, Germany). Directly after freeze-drying, 1–10 mg of freeze-dried sample was transferred into a 25-µL aluminum pan, sealed, weighed and placed in the DSC instrument. An empty pan was used as a reference sample. For analysis of glass transitions, samples were cooled to −20 °C followed by heating to 120 °C, cooling to −20 °C and heating to 150 °C; using a rate of 10 °C min^−1^. The first scan was used to obtain a uniform sample, and actual glass transition temperatures (T_g_ values) were determined from the second heating scan using Netzsch Proteus Analysis software. The T_g_ was taken as the onset temperature of the glass transition.

### Fourier transform infrared spectroscopy studies

A Perkin-Elmer 100 Fourier transform infrared (FTIR) spectrometer (Perkin-Elmer, Norwalk, CT, USA) was used to record infrared spectra. The spectrometer was equipped with a triglycine sulfate (TGS) detector, and an attenuated total reflection (ATR) accessory with diamond/ZnSe crystal and pressure arm. A temperature-controlled sample holder was available, for acquisition of transmission spectra. This was connected to a heating device (Harrick Scientific Products, Pleasantville, NY) and a Linkam pump system using liquid nitrogen as coolant (Linkam Scientific Instruments, Tadworth, Surrey, UK). The optical bench was continuously purged with dry air from an FTIR purge gas generator (Whatman, Clifton, NJ, USA). Spectra acquisition parameters were: 4 cm^−1^ resolution, 4 co-added interferograms, and 4000–900 cm^−1^ wavenumber range.

To study glassy behavior of freeze-drying formulations, mixtures of glucose, sucrose or trehalose and albumin were prepared at different mass ratios and a final concentration of 20–50 mg mL^−1^. Ten to twenty μL of a sugar/albumin solution was transferred onto a CaF_2_ window and dried under a stream of dry air (relative humidity less than 3%) to form sugar glasses. Residual water was removed by heating the sample to 100 °C, for 3 min, after which samples were cooled to −30 °C followed by heating to 180 °C at 1 °C min^−1^. Spectra were collected every 30–60 s. The band position of the OH-stretching vibration at ~3300 cm^−1^ (νOH) was monitored versus the sample temperature to follow glassy behavior, as previously described^28, 49^. By plotting νOH as a function of the sample temperature and adding linear regression lines in both liquid and glassy state regions; T_g_ was determined as the intersection point of these two regression lines.

To study membrane phase behavior of fresh and rehydrated cells, 10 μL cell pellets were used. Samples were cooled down to 0 °C followed by heating up to 80 °C at 2 °C min^−1^, while spectra were acquired every 30–60 s. The band position of the symmetric CH_2_ stretching vibration band arising from the lipid acyl chains at ~2850 cm^−1^ (νCH_2_) was followed versus the sample temperature, as described in detail elsewhere^50^. Membrane phase transitions were determined after taking first derivatives of the νCH_2_ versus temperature plots, using a 20-point smoothing factor.

The overall protein secondary structure of hydrated and dried cell samples was studied using ATR-FTIR. To reduce the contribution of interfering water bands, hydrated samples were (re)suspended in saline/D_2_O. Saline/D_2_O was obtained via adding D_2_O to freeze-dried samples. Cell samples were incubated for 1 hour in saline/D2O prior to measuring. The 1700–1600 cm^−1^ spectral region was selected to analyze the overall protein secondary structure of the samples. Second derivatives were taken, using a 13-point smoothing factor, and spectra were normalized to resolve differences in peak intensities more clearly. For comparison, denatured samples, prepared by incubation for 10 min at 80 °C, were also analyzed. To quantify the overall similarity between two second-derivative spectra, the correlation coefficient (r) was calculated using the following equation^51^:

$${\rm{r}}=\frac{{\sum }^{}{x}_{i}{y}_{i}}{\sqrt{{\sum }^{}{{x}_{i}}^{2}{\sum }^{}{{y}_{i}}^{2}}}$$

where x_i_ and y_i_ represent the spectral absorbance values of the reference and sample spectra at frequency position i. For identical spectra, a value of 1.0 will be returned. Spectra that have differences will show lower values.

### Single cell gel electrophoresis or ‘comet assay’ to study DNA damage

Samples after freeze-drying were carefully closed with stoppers and added in vacuum-sealed bags. Samples were stored at 4, 22 or 37 °C for up to 28 d. After storage for different durations, samples were transferred to −150 °C for later rehydration and analysis of multiple samples at once. To evaluate chromatin integrity and DNA damage, single cell gel electrophoresis (SCGE) was used. This assay is also known as the ‘comet assay’ and is described in detail elsewhere^52, 53^. In short, cells were embedded in agarose on microscope slides, and subjected to electrophoresis in alkaline conditions to separate DNA fragments of different sizes from the nucleus. Microscope slides were first cleaned (with 0.25 M HCl in 70% ethanol), coated with 0.5% (w/v) agarose and dried for 24 h at 37 °C. Slides were stored at room temperature until use. For SCGE, cell suspensions (50 μL ~20 × 10^6^ cells mL^−1^) were diluted in 1% agarose in saline of ~37 °C (800 μL), resulting in 1 × 10^6^ cells mL^−1^. Then, on a plate set at 37 °C, two 14 µL droplets were added per agarose-coated slide which were directly covered with a coverslip (10 × 10 mm). The slides were transferred to a pre-cooled shelf at 4 °C, and incubated for 7 min. After solidification of the agarose, the coverslips were carefully removed. Further procedures were carried out under dimmed light, and solutions were kept at 4 °C. Specimens were treated with lysis solution (2.5 mM NaCl, 0.1 M Na_2_EDTA, 10 mM TRIS, 0.1% Triton-X100, 25 mM DTT), for 30 min at 4 °C. DTT was added to the lysis solution just before use. Slides were kept in horizontal position, and 1 mL solution was added on each slide. After incubation, the lysis solution was removed, and specimens were treated with alkaline solution (300 mM NaOH, 1 mM Na_2_EDTA, pH > 13), for 30 min at 4 °C. After this, electrophoresis was performed in alkaline solution of 4 °C for 20 min at 20 V and 300 mA, using a ‘Comet Plus’ electrophoresis unit (Biostep GmbH, Burkhardtsdorf, Germany) with power supply (Biometra GmbH, Göttingen, Germany). Care was taken to position all slides in the same orientation. After electrophoresis, staining jars were used to wash slides in distilled water, and dehydration via exposure to a graded ethanol series (70, 90, and 100 v-%; 2 min each). After this, samples were air-dried. After drying, 10 µL 150 μg mL^−1^ Hoechst33342 staining solution was added on the agarose with embedded cells, a coverslip was added and specimens were sealed using nail polish. Specimens were observed using a fluorescence microscope (BX60; Olympus Corporation, Tokyo, Japan) equipped with ‘Colorview II’ camera and accompanying ‘Cell D’ software (Olympus Corporation). Micrographs were collected using at a 10 × 10 magnification and 2 s exposure time, and a minimum of 30 cells/‘comets’ were analyzed per treatment per independent experiment using ‘Komet’ software (Andor Technology Ltd, Belfast, UK). For preliminary experiments, ‘CaspLab’ was used for analysis of comets^54^.

### Statistical analysis

Statistical analysis was done using ‘Sigmaplot’ software (version 13; Systat Software Inc., San Jose, CA). The data points presented in this study represent mean values from 3–4 independent experiments, and error bars denote the standard deviation. To determine if differences between treatments/experimental groups were significant, one-way analysis of variance (ANOVA) was performed. Differences were taken to be statistically significant in case p < 0.05.
